# Supplementary material for: In Silico Prediction of Skin Permeability Using a Two-QSAR Approach
Source: Pharmaceutics. 2022 Apr 28;14(5):961. doi: 10.3390/pharmaceutics14050961 (PMC9143389; doi:10.3390/pharmaceutics14050961)
Supplement: Supplementary file 1 [file pharmaceutics-14-00961-s001.zip › Figure S1.pdf]

In Silico Prediction of Skin Permeability Using a Two-QSAR Approach

Supporting information

Yu-Wen Wu[1,#], Giang Huong Ta[1,#], Yi-Chieh Lung[1], , Ching-Feng Weng[2] and Max K. Leong[1,*]

[1] Department of Chemistry, National Dong Hwa University, Shoufeng, Hualien 974301, Taiwan
[2] Department of Basic Medical Science, Center for Transitional Medicine, Xiamen Medical College, Xiamen 361023, Fujian, China
* Correspondence: leong@gms.ndhu.edu.tw; Tel.: +886-3-890-3609
[#] Equal contribution as first authorship

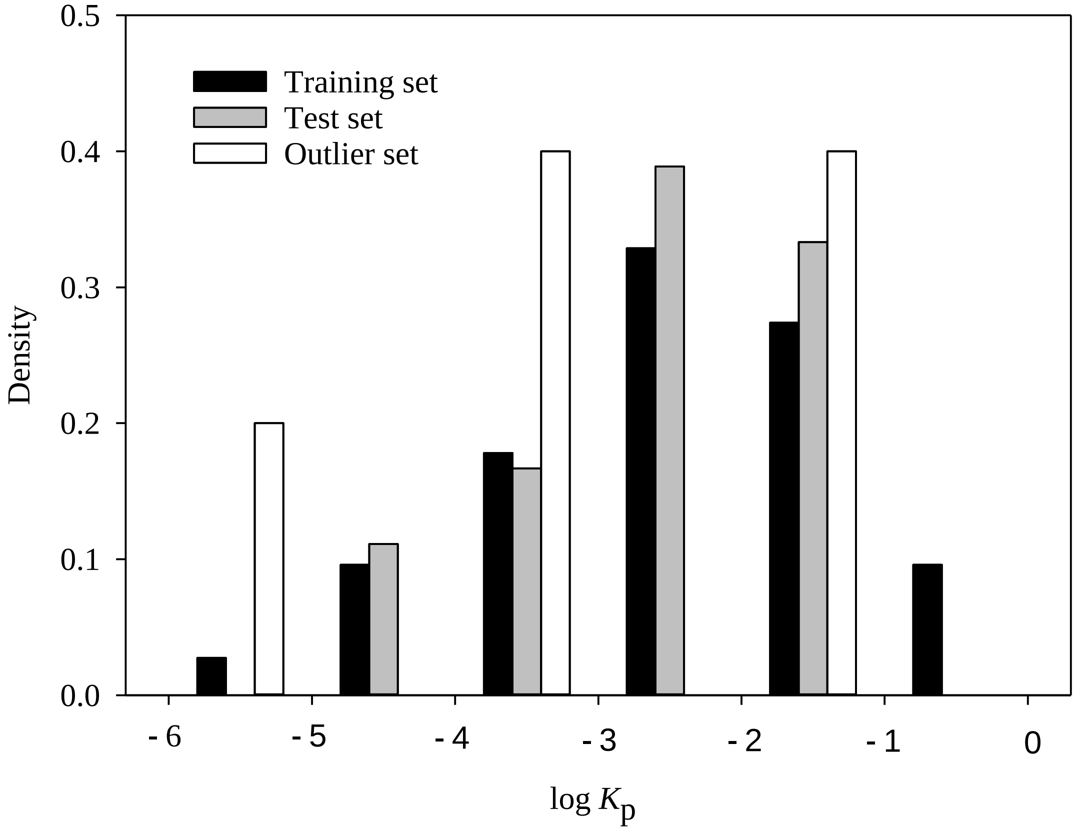

(**A**) Histogram representation of the log K_P distribution for all molecules in the training set, test set, and outlier set.

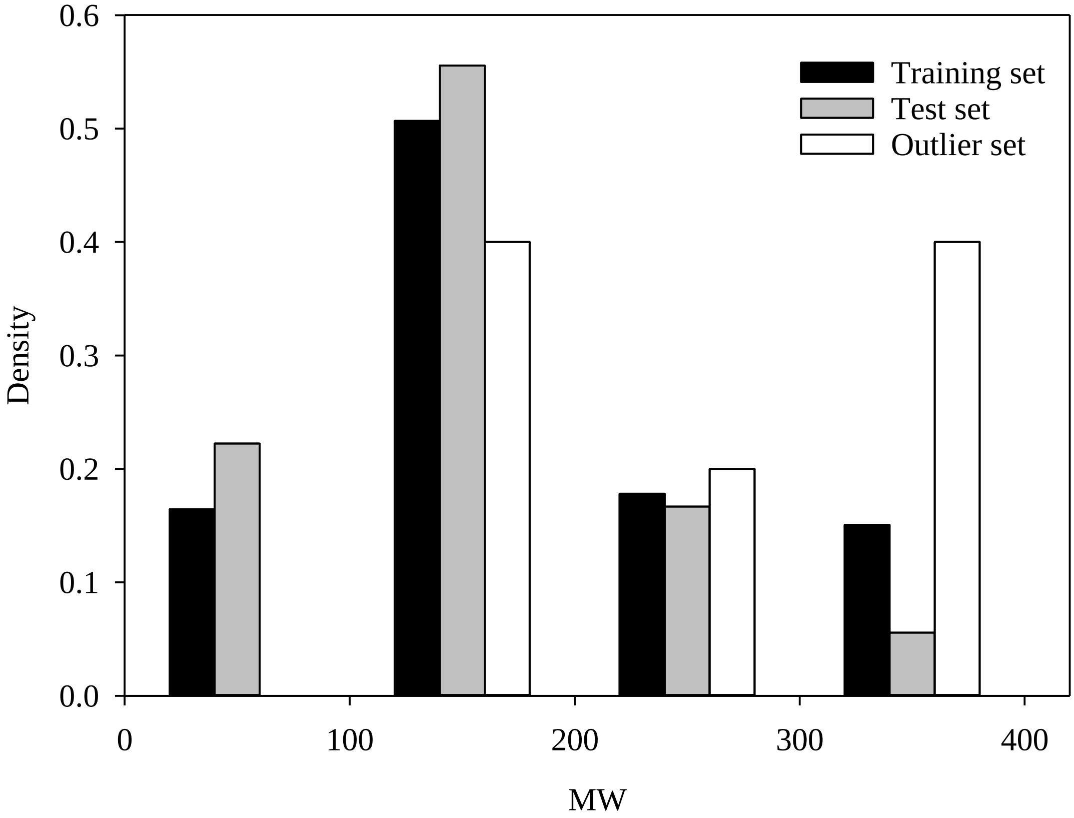

(**B**) Histogram representation of the molecular weight (MW) distribution for all molecules in the training set, test set, and outlier set.

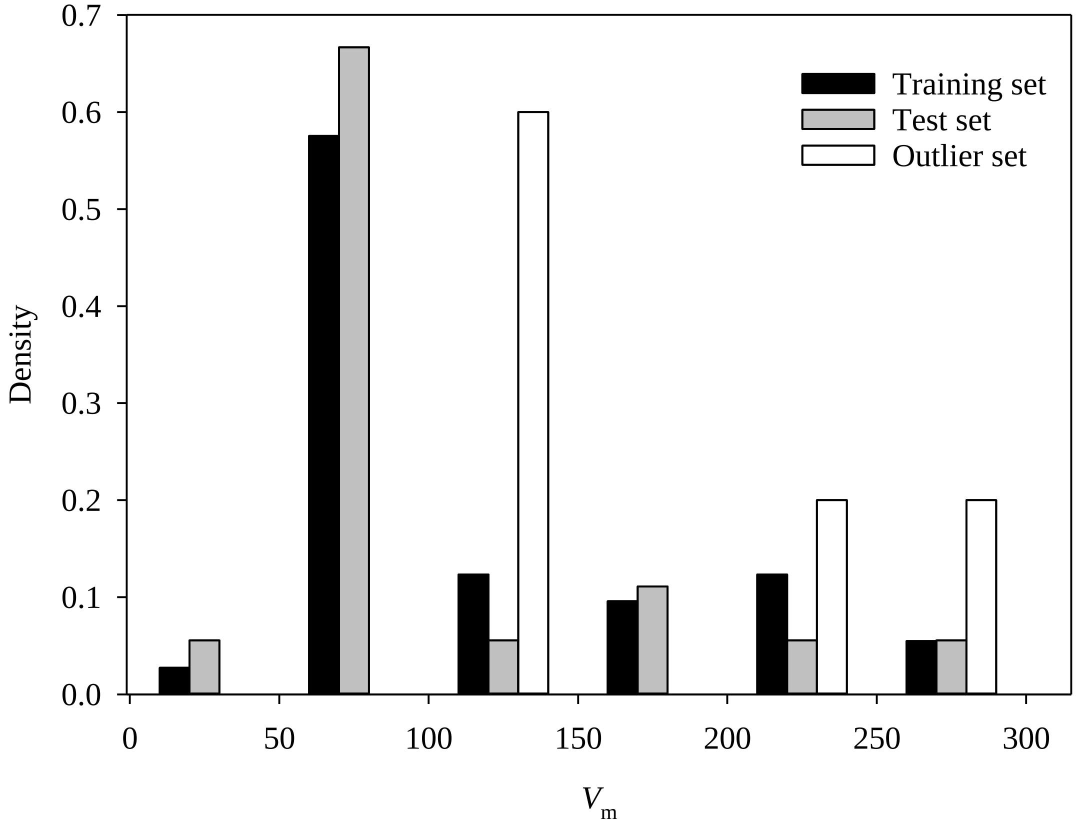

(**C**) Histogram representation of the molecular volume (V_m) distribution for all molecules in the training set, test set, and outlier set.

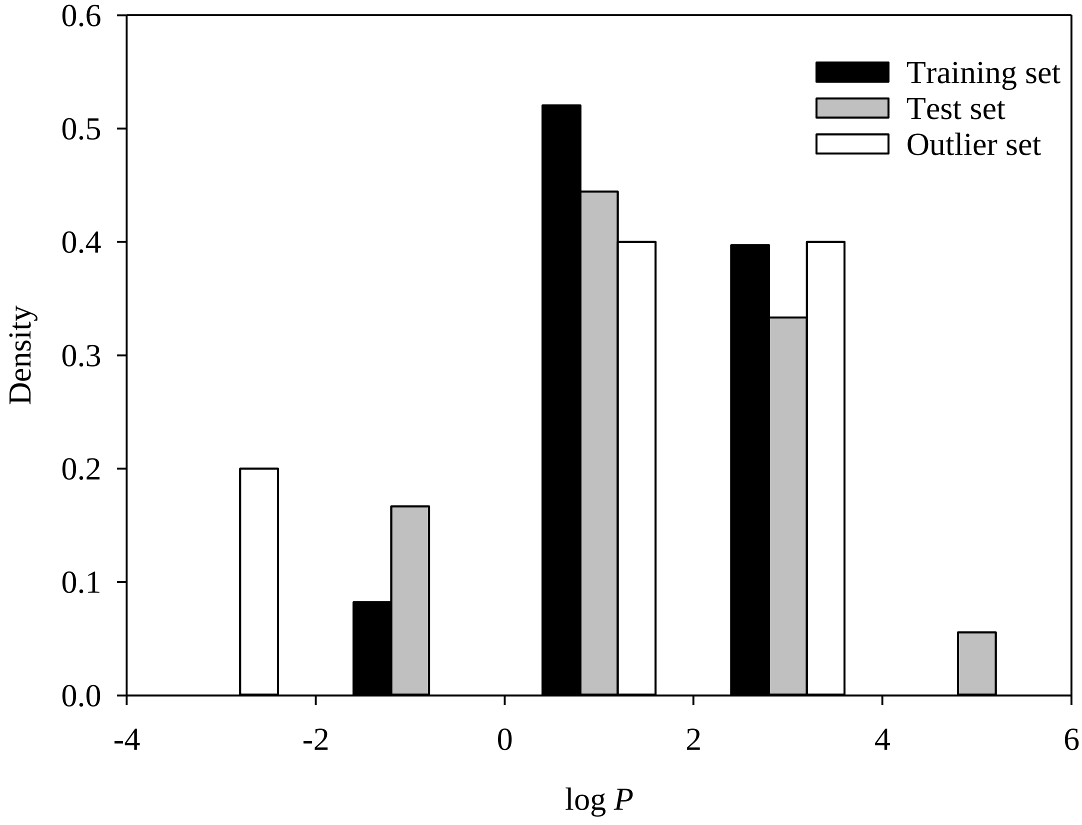

(**D**) Histogram representation of the *n*-octanol–water partition coefficient (log *P*) distribution for all molecules in the training set, test set, and outlier set.

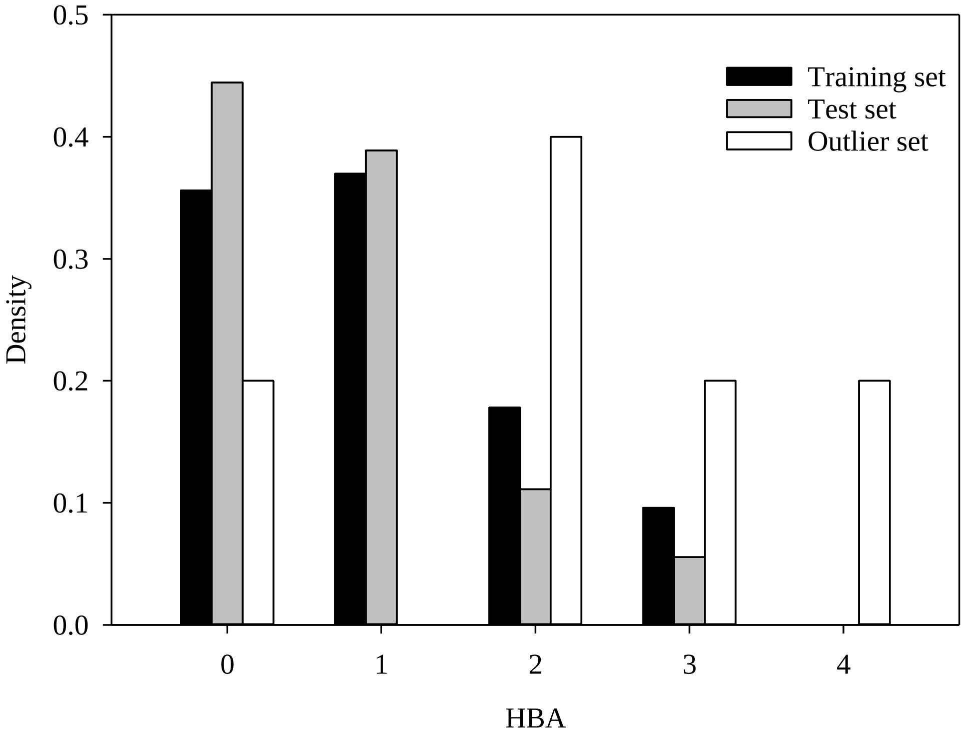

(**E**) Histogram representation of the number of hydrogen bond acceptor (HBA) distribution for all molecules in the training set, test set, and outlier set.

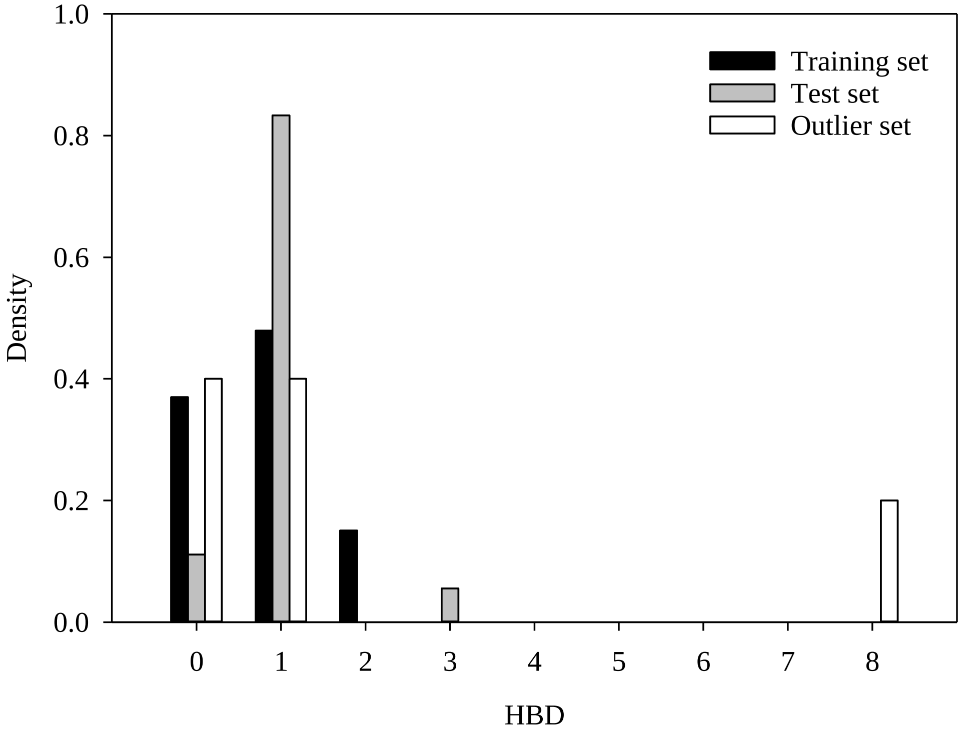

(**F**) Histogram representation of the number of hydrogen bond donor (HBD) distribution for all molecules in the training set, test set, and outlier set.

Figure S1. Histogram representation of the distributions of various descriptors for all molecules in the training set, test set, and outlier set.
